# Bibliometric Analysis of the 100 Most-Cited Articles on Periodontics in the Arab World

**DOI:** 10.7759/cureus.45734

**Published:** 2023-09-21

**Authors:** Raghad S Aljabbary, Ikram Ul Haq, Sohaib Shujaat

**Affiliations:** 1 College of Dentistry, King Saud Bin Abdulaziz University for Health Sciences, Riyadh, SAU; 2 Department of Oral and Maxillofacial Surgery, Katholieke Universiteit Leuven, Leuven, BEL; 3 Department of Maxillofacial Surgery and Diagnostic Science, King Abdullah International Medical Research Center, Riyadh, SAU

**Keywords:** periodontology, research productivity, bibliometrics, arab countries, periodontics

## Abstract

Aim: Periodontics is a branch of dentistry that deals with diseases of the supporting and investing structures of the teeth including the gingiva, cementum, periodontal ligament, and alveolar bone. The current study aimed to scrutinize the bibliometric indicators of the 100 most-cited articles on periodontics contributed by authors affiliated with the Arab countries.

Research methodology: The bibliometric research method was used for the 100 most-cited articles retrieved from the Scopus database on May 9, 2023. The keywords *periodontitis*,* periodontology*,* gingivitis*,* periodontal*,* gingiva,* and *periodontium *were inserted, and then the Arab countries were selected from the country’s filter. The 100 most-cited articles were accessed. The bibliometric indicators such as periodic distribution of articles, their study design, nature of research, contributed by different Arab countries, international research collaboration, and author’s used keywords were analyzed. Microsoft Excel version 16 (Microsoft Corporation, Washington, United States), VOSviewer (Centre for Science and Technology Studies, Leiden University, The Netherlands), and SPSS Statistics version 27 (IBM Corp. Released 2020. IBM SPSS Statistics for Windows, Version 27.0. Armonk, NY: IBM Corp.) were used for data analysis.

Results: A slow progress (4.50%) was found in periodontics literature before 2000 in the Arab countries, but a significant growth (75%) was recorded during the past 10 years (January 1, 2014, to May 9, 2023). The 100 most-cited articles on periodontics by the Arab countries were published from 1995 to 2021, and these articles gained citations with an average of 92.18 citations per articles. *Case-control* and *review* studies were the preferred study designs, and *periodontology* and *implantology* were the top two subject categories. About one-third of the articles were published in the *Journal of Periodontology *and the *Journal of Clinical Periodontology*. Saudi Arabia contributed the highest number of the most-cited articles, followed by Egypt and Jordan, but the articles contributed by the United Arab Emirates were the most influential. The United States and Germany were the top-ranked countries in international research collaboration.

Conclusion: The findings demonstrate that periodontics research increased remarkably during the past 10 years. Saudi Arabia stands not only on the topmost rank in overall research productivity in the Arab countries but also surpasses the rest of the Arab countries in the 100 most-cited articles. Saudi Arabia contributed 26 articles with the United States, while Jordan contributed the highest ratio of indigenous research.

## Introduction and background

The Arab World is a group of 22 countries (Algeria, Bahrain, Comoros, Djibouti, Egypt, Iraq, Jordan, Kuwait, Lebanon, Libya, Mauritania, Morocco, Oman, Palestine, Qatar, Saudi Arabia, Somalia, Sudan, Syria, Tunisia, United Arab Emirates, and Yemen), whose first language is Arabic, and all these countries are the members of the Arab League that was formed in 1945 [1]. The Arab countries have had a rich history of learning, research, and exploration for many centuries, and Muslim scholars have contributed to the advancement of science. After centuries of neglect, scholarship was revitalized and institutionalized after the decolonization of numerous Arab governments. The majority of modern universities and research institutions were founded in the 1970s and 1980s in the Arab countries, resulting in visible scientific output [2].

Periodontics is a branch of dentistry that deals with diseases of the gingiva and connective tissue surrounding the teeth [3]. Periodontitis is one of the causes of tooth loss, and 90% of people have periodontal and gingival disorders. Many chronic diseases such as diabetes and renal and cardiovascular diseases may have a close connection to poor periodontal health [3]. Scientific research plays an important role in introducing novel treatment techniques and sharing the findings to expand the boundaries of research [4]. Owing to continuous research, periodontitis has become a readily treatable and preventable disease [5].

The evaluation of research growth is of paramount importance, and bibliometric analysis serves as an integral instrument in gauging research performance. The bibliometric quantitative technique has been applied as an instrument to analyze previously published studies [6]. This method can be used at various levels, from the scholarly work of individual authors to the collective research profile of institution(s), from national to regional and even global levels [7]. Based on the citation metrics, the bibliometric analysis provides the dataset on the most-cited article in a particular knowledge domain that helps to understand the research trends [8]. The articles extensively cited by the peer researchers in their scholarly work are considered as one of the most-cited articles [9]. Citations possess the prospective ability to serve as indicators of a publication's impact within the context of an ever-expanding scientific literary landscape [10]. The assessment of the academic standing of researchers is often based on the number of citations that their work receives, as well as the impact factor of the journal in which their research is published [11].

A recent study analyzed PubMed-indexed dental articles produced by the Arab countries. “Oral surgery” was the preferred area of research succeeded by “orthodontics/pedodontics” and “conservative dentistry/endodontics.” Saudi Arabia emerged as the most productive among other Arab countries [12]. Another study also endorsed that about 40% of dental literature from 1998 to 2017 in the Arab countries was produced by Saudi Arabia [13]. Alfadley et al. scrutinized the scientific output on endodontics produced by the six Arab countries members of the Gulf Cooperation Council. This region contributed to 2.82% of the global endodontic research, and about 80% of the literature was produced by Saudi Arabia [14]. Bennani et al. studied the dental research contributed by the 66 dental institutions located in 19 countries of Africa [15]. About one-third (32%) of the literature was produced in Egypt followed by South Africa and Nigeria. The highest number of articles were written on “conservative dentistry” followed by “periodontology” [16].

Researchers have been conducting bibliometric studies on different areas of knowledge in the Arab World. El Rassi et al. assessed the research output in medical sciences in the Arab World. Egypt, Saudi Arabia, and Tunisia produced 32%, 28%, and 11% of the literature, respectively. The maximum number of research was produced by King Saud University followed by Cairo University [16].

Feijoo et al. analyzed the 100 most-cited articles in dentistry. The study found that 43% of the articles fell in the sub-category of periodontology and 66% of the articles belonged to clinical types of study [17]. Recently, Asiri et al. also scrutinized the 100 most-cited articles on dentistry. About half of the articles (48%) were contributed by the United States, and periodontology was the most occurred area of interest, and 36% of the articles were written as “narrative reviews” followed by “clinical trials” [9].

Alarcón et al. examined the top 300 most-cited articles on periodontology. The analysis of study type revealed that narrative review (n=81) and cross-sectional (n=71) studies were very common, but the controlled clinical trial (n=13) gained the highest mean value of citations [18]. Nieri et al. evaluated the citation of classic articles on periodontology and compared it with the less cited articles. The article cited at least 100 times considered as classic. The classic articles were longer, used more figures/tables, and had more authors [19]. Ababneh et al. gauged the publications on periodontics contributed by Saudi Arabia. Saudi Arabia contributed 4.40% of the global periodontics research output, and the highest research collaboration was done with the United States [20]. Alrubaig et al. reported that Saudi Arabia contributed 3.29% of the global endodontic research. The articles with international research collaboration gained more citations as compared to indigenous research [21].

Although some studies focused on dental research in the Arab countries, the area of most-cited articles on dentistry and its sub-category was not explored. The current study was intended to fill this knowledge gap. We used a bibliometric method to evaluate the salient features of the 100 most-cited articles on periodontics contributed by authors affiliated with the Arab countries.

## Review

Materials and methods

The quantitative bibliometric research method was applied to the 100 most-cited articles on periodontics produced by authors affiliated with the Arab World. The data was extracted on May 9, 2023, using the Scopus database.

In the first phase, the following keywords were used to extract relevant articles, periodontitis, periodontology, gingivitis, periodontal, gingiva, and periodontium.

In the second phase, 22 Arab countries were selected from the country filter to limit our search, but we found only 19 countries (Saudi Arabia, Egypt, Iraq, Jordan, United Arab Emirates, Kuwait, Lebanon, Morocco, Libya, Tunisia, Syrian Arab Republic, Qatar, Yemen, Sudan, Oman, Palestine, Bahrain, Algeria, and Djibouti) with research productivity ranging from one article to 48 articles. The authors affiliated with Mauritania, Somalia, and Comoros didn’t contribute any articles on periodontics in Scopus-indexed journals. The overview of scholarly literature on periodontics by the Arab world was evaluated.

**Figure 1:**
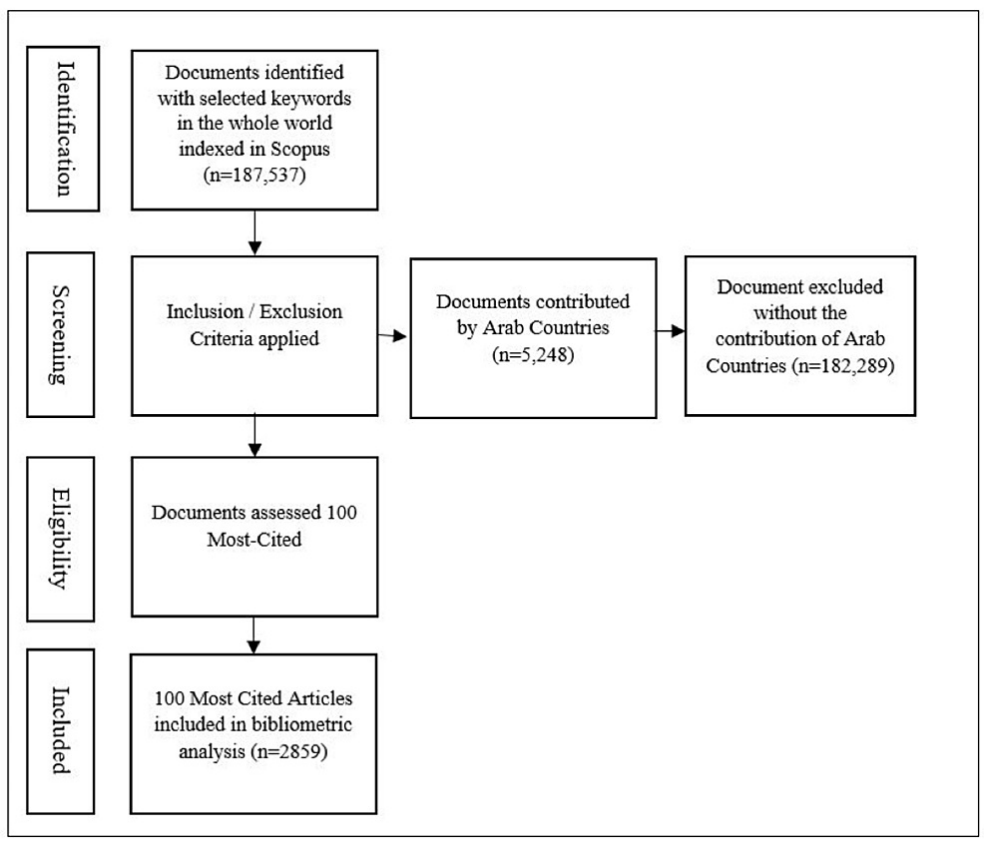
Screening process of the 100 most-cited articles in the Scopus database

In the third phase, the 100 most-cited articles were selected, and complete bibliographic records with abstract and citation metrics were downloaded in Excel format (Figure 1). The periodic growth of the articles and citation metric was assessed. These articles were thoroughly reviewed to identify their study design, nature of study (clinical and non-clinical), and sub-category of periodontics. The preferred source of publications contributed and the impact of periodontics research by the Arab countries, collaborative countries, and author’s used keywords were examined. Microsoft Excel version 16 (Microsoft Corporation, Washington, United States), VOSviewer (Centre for Science and Technology Studies, Leiden University, The Netherlands), and SPSS Statistics version 27 (IBM Corp. Released 2020. IBM SPSS Statistics for Windows, Version 27.0. Armonk, NY: IBM Corp.) were used for data analysis.

Results

**Figure 2:**
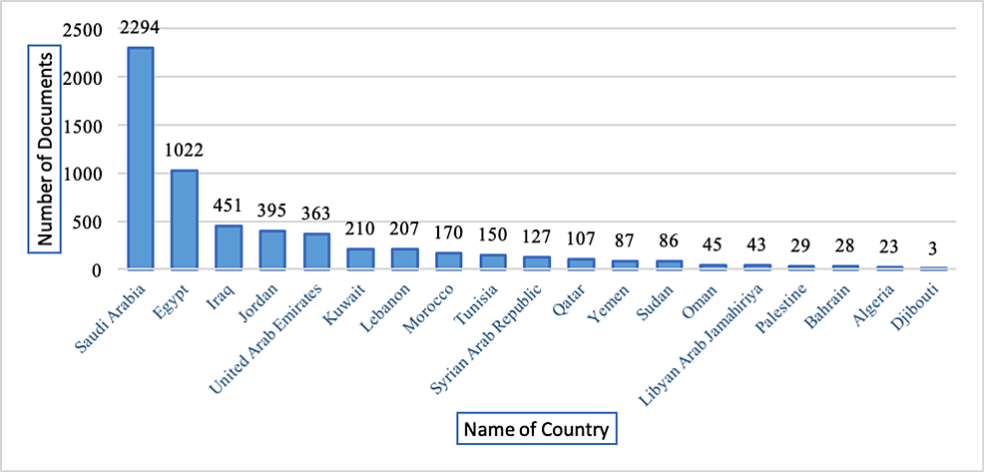
Distribution of documents by the Arab countries (n=5243)

The Arab countries contributed 5,243 documents on periodontics from 1948 to May 2023, and only 236 (4.50%) documents were published in the first 53 years from 1948 to 2000. Three-fourths of the documents (n=3926; 74.88%) were published during the last 10 years from January 1, 2014, to May 9, 2023. Figure 2 presents the highest number of documents contributed by Saudi Arabia (n=2,294; 43.75%), followed by Egypt (n=1,022; 19.49%) and Iraq (n=451; 8.60%), while Djibouti contributed the lowest number of documents (n=3).

The analyses of international research collaboration with the Arab Countries showed that the United States was the most preferred option for research collaborations (n=774; 15%) followed by India (n=514; 9.80%), the United Kingdom (n=311; 5.93%), Pakistan (n=197;3.75%), and Germany (n=174; 3.31%). The analyses of documents at the institutional level revealed that King Saud University, Saudi Arabia, appeared to be the most productive institution with 684 documents, followed by King Abdulaziz University, Saudi Arabia (n=329), Cairo University, Egypt (n=323), Jordan University of Science and Technology, Jordan (n=200), Jazan University, Saudi Arabia (n=199), and Imam Abdulrahman bin Faisal University, Saudi Arabia (n=168).

About 60% (n=3,084) of the documents were published in 117 journals, having 10 or more than 10 documents each. The maximum number of documents were published in the Journal of Periodontology (n=137), followed by the Journal of Contemporary Dental Practice (n=125), the Saudi Dental Journal (n=108), and BMC Oral Health (n=91).

The analysis of document types showed that articles consisted of 83.35% (n=4370), followed by review (n=636; 12.13%) and other documents (book chapter, letters, conference paper, editorial, erratum, note, short survey, book, and retracted) 237 (4.52%).

**Figure 3:**
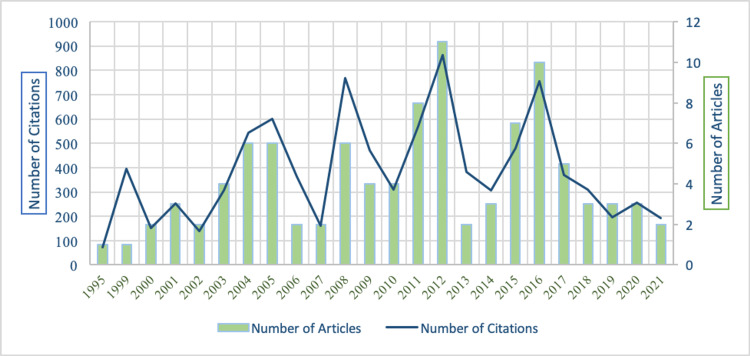
Periodic growth of articles by years with citation metrics

Out of 5,243 documents, the 100 most-cited articles on periodontics were selected for analysis. These articles were published in the span of 27 years from 1995 to 2021. The study divided 27 years into three equal intervals of nine years each. During the first interval (1995-2003), 13 articles were published, and these articles were cited 1,318 times with an average of 101.38 citations per article. The highest number of articles (n=49) were published in the second interval (2004-2012), and these articles gained an average of 94.89 citations per article. In the last interval (2013-2021), 38 articles were published, and these articles gained comparatively low citation impact (85.52 cites/article). Overall, the 100 most-cited articles gained 9,218 citations with an average of 92.18 cites/article. The highest number of most-cited articles (n=12) were published in the year 2012, followed by 2016 and 2011 with 10 and eight articles, respectively. The highest citation impact (128.16 cites/article) was received from six articles published in 2006, and the lowest citation impact (65.33 cites/article) was gained from three articles published in 2019 (Figure 3).

The ratio of clinical studies was found higher than non-clinical articles (57:43), but non-clinical articles gained a higher citation impact (99.18 cites/article) as compared to clinical articles (86.89 cites/article). A chi-square test was conducted as part of the descriptive statistical analysis, yielding a p-value of 0.51, which showed that no statistically significant difference existed between the number of articles and citations in clinical and non-clinical studies (Table 1).

**Table 1:** Distribution of articles based on clinical and non-clinical categories

Format of study	Articles	Citations	Citation impact	Statistical test
Clinical	57	4,953	86.89	p-value 0.51 X^2^=0.47
Non-clinical	43	4,265	99.18	

Study design

The assessment of the most-cited articles by study design is summarized in Table 2. Nineteen different study designs had been used by the authors in the most-cited articles. The case-control study design was found to be the most frequent (n=21), followed by review (n=13) and randomized controlled trials (n=10). Apart from one article each in the last six study designs, four articles fell in the study design of meta-analysis and received the highest citation impact (170.25 cites/article), followed by systematic review and meta-analysis (116.67 cites/article). Comparative study design gained the lowest citations (71.50 cites/article).

**Table 2:** Distribution of most-cited articles by study design

Serial no	Study design	Total articles	Total citations	Citation impact
1	Case-control study	21	1,885	89.76
2	Review	13	1,197	92.08
3	Randomized controlled trial	10	854	85.40
4	Comparative study	8	572	71.50
5	Cross-sectional	7	672	96.00
6	Clinical trail	7	481	68.71
7	Systematic review and meta-analysis	6	700	116.67
8	Systematic review	6	473	78.83
9	In vitro	5	417	83.40
10	Meta-analysis	4	681	170.25
11	Prospective longitudinal cohort study	3	428	142.67
12	Laboratory study	2	175	87.50
13	Animal study	2	147	73.50
14	Ecological study	1	132	132.00
15	In vitro/in vivo	1	92	92.00
16	Double-blind randomized controlled trial	1	81	81.00
17	Retrospective study	1	81	81.00
18	Scanning electron microscope study	1	80	80.00
19	Prospective, two-center study	1	70	70.00

Table 3 presents the details of articles by sub-category of periodontics. The 100 most-cited articles were distributed into 14 sub-categories, and most of the articles were published in periodontology (n=47), followed by implantology (n=17), risk factors of periodontal diseases (n=8), and non-surgical periodontal therapy (n=8). Apart from one article each in the last six sub-categories, the highest citation impact (140.80 cites/article) was received by five articles on systemic disease and periodontal health. Non-surgical periodontal therapy gained the lowest citation impact (69 cites/article).

**Table 3:** Distribution of articles by sub-category of periodontics

Rank	Sub-category	Total articles	Total citation	Citations impact
1	Periodontology	47	3,573	76.02
2	Implantology	17	1,713	100.76
3	Risk factors of periodontal diseases	8	809	101.13
4	Non-surgical periodontal therapy	8	552	69.00
5	Systemic diseases and periodontal health	5	704	140.80
6	Epidemiology of periodontal diseases	4	331	82.75
7	Tissue engineering and Periodontal regeneration	3	396	132.00
8	Microbial etiology of periodontal diseases	2	360	180.00
9	Pathogenesis of periodontal diseases	1	396	396.00
10	Antibiotics in periodontology	1	103	103.00
11	Genetics	1	84	84.00
12	Histology	1	78	78.00
13	Dental biofilm	1	60	60.00
14	Periodontium	1	59	59.00

All the top-cited articles were published in 51 journals, and 62 articles were published in 13 journals listed in Table 4, having two or more than two articles. There were 38 journals with one article each. The highest number of articles were published in the Journal of Periodontology (n=23), followed by the Journal of Clinical Periodontology (n=9). As per citation impact, Clinical Implant Dentistry and Related Research ranked at the top (116 cites/article), followed by the International Journal of Oral and Maxillofacial Implants (114 cites/article) and the Journal of Periodontology (100.87 cites/article).

**Table 4:** Top 13 journals having more than one article each

Ranks	Name of journal	Total articles	Total citations	Citation impact
1	Journal of Periodontology	23	2,320	100.87
2	Journal of Clinical Periodontology	9	712	79.11
3	International Journal of Oral and Maxillofacial Implants	4	456	114.00
4	Journal of Periodontal Research	4	282	70.50
5	Photodiagnosis and Photodynamic Therapy	4	268	67.00
6	Journal of Dental Research	3	228	76.00
7	Journal of Oral Rehabilitation	3	194	64.67
8	Clinical Implant Dentistry and Related Research	2	232	116.00
9	AAPS PharmSciTech	2	174	87.00
10	Disease Markers	2	135	67.50
11	Clinical Oral Implants Research	2	135	67.50
12	Archives of Oral Biology	2	130	65.00
13	Implant Dentistry	2	113	56.50

In Table 5, only 10 Arab countries contributed to the most-cited 100 articles on periodontics, and Saudi Arabia contributed the maximum number of articles (n=48), followed by Egypt and Jordan with 18 and 16 articles, respectively. Although the United Arab Emirates and Morocco published three and two articles, respectively, these articles gained the higher citation impact with 190 and 170 cites/article.

**Table 5:** Contribution and impact of Arab countries in most-cited articles

Serial no	Arab country	Total articles	Citations	Citation impact
1	Saudi Arabia	48	4,013	83.60
2	Egypt	18	1,694	94.11
3	Jordan	16	1,527	95.44
4	Kuwait	7	567	81.00
5	Morocco	3	522	174.00
6	Lebanon	3	277	92.33
7	United Arab Emirates	2	380	190.00
8	Qatar	2	216	108.00
9	Syrian Arab Republic	2	131	65.50
10	Iraq	1	54	54.00

The authors affiliated with the 10 Arab countries collaborated with the authors of 25 countries of the world in the most-cited 100 articles on periodontics. More than one-third (n=36) of the articles were collaborated with the United States, followed by Germany (n=13) and Pakistan (n=8). Nine countries were found with one article each in collaboration. The analysis of impact based on citation count disclosed that the articles that were collaborated with Denmark had the highest citation impact, followed by Australia and the United Kingdom (Table 6).

**Table 6:** Research collaborative countries

Serial no	Arab country	Articles	Citations	Citation impact
1	United States	36	3,162	87.83
2	Germany	13	1,078	82.92
3	Pakistan	8	583	72.87
4	Malaysia	7	500	71.42
5	Spain	4	360	90.00
6	Sweden	4	374	93.5
7	United Kingdom	4	629	157.25
8	Australia	3	538	179.33
9	Canada	3	316	105.33
10	Greece	3	216	72.00
11	India	3	234	78.00
12	Italy	3	329	109.66
13	Brazil	2	127	63.50
14	China	2	137	68.50
15	Denmark	2	361	180.50
16	Norway	2	130	65.00
17	France, Hong Kong, New Zealand, Poland, Slovenia, South Korea, Taiwan, and Ethiopia	1 article each		

A total of 280 keywords were used by the authors, and the keyword “periodontitis” was used 23 times followed by "periodontal disease" (n=11). The keywords "meta-analysis" and "smoking" occurred seven times each (Table 7).

**Table 7:** Authors’ used keywords

Serial no	Keyword(s)	Total occurrence
1	Periodontitis	23
2	Periodontal disease	11
3	Meta-analysis	7
4	Smoking	7
5	Peri-implantitis	6
6	Chronic periodontitis, oral hygiene, periodontal index	5 times each
7	Diabetes mellitus, periodontal regeneration, photodynamic therapy, risk factors, tooth loss	4 times each
8	Alveolar bone loss, collagen, dental implant, dental implants, diabetes, gingiva, GTR, implant, inflammation, obesity, periodontal, periodontal pocket, *Salvadora persica*, stem cells, type 2 diabetes mellitus	3 times each
9	35 keywords	2 times each
10	217 keywords	One time each

Discussion

Citation metrics are used to assess the quality of published research. The greater the number of citations referred to, the greater the quality of the scientific work [22]. The citation metrics have frequently been used as a logical measure of an article's academic influence [23]. Citation analysis is one of the indicators of bibliometric studies, and it has been widely reported in the dental literature [24]. The current bibliometric study presents a range of findings that hold significance in revealing the research patterns of the 100 most-cited articles on periodontics in the Arab countries.

It was discovered that dental researchers from the Arab countries have produced 5,243 documents over seven decades in periodontics as indexed in the Scopus database. Despite an upward trend in the number of documents in the past decade (2014 to May 2023), it remains comparatively low. The evolution of periodontics literature showed that only 4.50% of documents on periodontics were published from 1948 to 2000. The potential explanations for the lack of periodontics literature before 2000 are that the Arab countries have very few dental institutions, less number of Scopus-indexed journals, and even less number of dental researchers [12].

Periodontics is an important branch of dentistry. The studies on the 100 most-cited articles on dentistry revealed that periodontology was the most frequently occurring branch of dentistry as Feijoo et al. reported that 43% of the articles fall in the category of periodontology and the highest number of the most-cited articles were published in the Journal of Clinical Periodontology [14]. Another study conducted by Asiri et al. on the 100 most-cited articles on dentistry also confirmed that most of the articles (26%) were related to periodontology [21]. A study examining PubMed-indexed dental articles contributed by the Arab countries showed that “oral surgery” was the preferred area of research (33.66%), succeeded by “orthodontics/pedodontics” (26.78%) [12].

The most-cited articles were published during the period of 27 years from 1995 to 2021, and these articles gained an average of 92.18 citations per article with a range of citations from a minimum of 54 to a maximum of 396 in our study. Grillo et al. analyzed the 100 most-cited articles on oral and maxillofacial literature produced by the 17 countries of the Middle East. These articles were published between 1990 and 2018 and gained a range of citations from a minimum of 67 to a maximum of 328 citations [25]. In our study, Saudi Arabia contributed the highest number of most-cited articles, whereas Turkey was found at the top among the Middle East countries. Nieri et al. evaluated the 55 classic articles on periodontology, and these articles were published from 1990 to 2005. These findings showed that the most-cited articles on periodontology started to be published during the 1990s [19]. Ahmed et al. inspected the 100 most-cited articles published in Periodontology 2000. These articles were published from 1993 to 2014 and gained an average of 212.76 citations per article. The 100 most-cited articles published in Periodontology 2000 gained more citations than the Arab countries’ contribution because Periodontology 2000 is one of the top-ranked journals in periodontology, and the high-quality research articles contributed by the best authors of the world have been published in this journal [26].

The analysis of study designs showed that “case-control study” was the most frequent in our study followed by “review” and “randomized control trial,” but the “meta-analysis” study design gained more citations. Alarcón et al. examined the 300 most-cited articles on periodontology and revealed that “narrative review” and “cross-sectional studies” were common, but the “controlled clinical trial” gained the highest mean value of citations [18]. Anas et al. stated that a “descriptive cross-sectional study” (31%) was common in dental institutions of the Arab countries, followed by “experimental in vitro study” and “literature review/systematic review” [12].

We divided the 100 most-cited articles into 14 sub-themes of periodontics, and “periodontology” was found to be the most preferred, followed by “implantology.” Ababneh et al. reported that “periodontitis” and “dental implants” were the most frequent topics in periodontics research in Saudi Arabia [20]. Another study of 300 most-cited articles on periodontics reported that “oral biology” and “detection of bacteria” were the top research themes [18].

About one-third (32%) of the most-cited articles were published in the Journal of Periodontology (n=23) followed, by the Journal of Clinical Periodontology (n=9). Ahmed and Slots stated that the Journal of Clinical Periodontology, Journal of Periodontology, and Periodontology 2000 are the three core journals of periodontics. Overall, the Arab countries contributed the highest number of articles (n=137) on periodontics published in the Journal of Periodontology [5].

Our study showed that an average of 5.29 authors contributed to the 100 most-cited articles, and only five articles were found with a single author, while the three-author pattern was found most frequently with 21 articles. Asiri et al. reported that of the 100 most-cited articles on dentistry, 20 articles were written by a single author, and 27 articles were written by two authors [9]. Nieri et al. assessed the citation of classic articles on periodontology and found that the most-cited articles had more authors as compared to less-cited articles [19].

Out of 22 Arab countries, only 10 countries contributed to the 100 most-cited articles, and the authors affiliated with Saudi Arabia contributed the highest number of articles (n=48), followed by Egypt (n=18) and Jordan (n=16). A study focused on dental research of the Arab countries indexed in PubMed stated that Saudi Arabia, Egypt, and Jordan produced about two-thirds of the total dental research [12]. Haq et al. examined the Scopus-indexed dental research of the Arab countries from 1998 to 2017. This study testified that Saudi Arabia contributed 40% of the research followed by Egypt and Jordan [13].

Limitations

This study has some limitations. Firstly, the data source was limited to a single database. Hence, importing data from other data sources, like Web of Science and PubMed, might give different citation metrics and findings. Furthermore, there were some inconsistencies related to the search strings in the Scopus database, and some articles might have been excluded because they did not match our search criteria.

The Scopus database provides comprehensive coverage of scholarly literature and citation counts and indexed more journals than the Web of Science and PubMed databases. The majority of the Web of Science and PubMed-indexed journals have been indexed in the Scopus database [27]. Google Scholar is basically a fragment of the World Wide Web search engine that also indexes the substandard material [27]. For the analysis of the most-cited articles, reliance on a single database is the preferred choice, otherwise, the study becomes a comparative analysis of citation counts in different databases. We have not exempted the self-citations of authors and journals that may inflate the ratio of citations.

For future studies, periodontics articles published from both Scopus and non-Scopus-indexed journals would bring a more comprehensive scenario of research productivity from the Arab countries.

Secondly, the number of researchers from the Arab countries who are currently engaged in research and academic pursuits in Europe, the United States, and other parts of the world is unknown. Our search query in the Scopus database depended on affiliation address as one of the authors must be affiliated with one of the Arab countries. The dental institutions and dental societies of the Arab countries should come forward with dental research policies, and the native dental researchers should invite other Arab researchers residing abroad for research collaborations.

Thirdly, we didn’t highlight the productive authors and institutions; future studies should consider these variables in their research.

Despite these limitations, few recommendations for future studies have been pinpointed, and this study offers an understanding of how most-cited articles related to periodontics in the Arab countries have been disseminated and occurred. The findings of this study may be beneficial for upcoming researchers in identifying top journals, collaborative research patterns, and subject dispersion as a benchmark for future studies.

## Conclusions

The findings demonstrated that periodontics research increased remarkably during the past 10 years in the Arab countries. The ratio of citation metrics by the Arab countries was found to be low compared to the most-cited articles in various specialties at a global level. Periodontology and implantology were identified as predominant fields of research. The authors from only 10 Arab countries contributed to the most-cited articles, and about half of the articles were contributed by Saudi Arabia. The highest ratio of collaboration was conducted between Saudi Arabia and the United States. These findings support the fact that periodontics researchers from the Arab countries should step up and take obligatory actions to increase the visibility of scholarly research.

## Appendices

**Table 8:** List of the 100 most-cited articles on periodontics in the Arab World

Serial no	Detail of articles	Citations
1	Toomes C, James J, Wood AJ, et al. Loss-of-function mutations in the cathepsin C gene result in periodontal disease and palmoplantar keratosis. Nat Genet. 1999;23(4):421-424. 10.1038/70525	396
2	Atieh MA, Alsabeeha NH, Faggion CM Jr, Duncan WJ. The frequency of peri-implant diseases: a systematic review and meta-analysis. J Periodontol. 2013;84(11):1586-1598. 10.1902/jop.2012.120592	325
3	Khader YS, Dauod AS, El-Qaderi SS, Alkafajei A, Batayha WQ. Periodontal status of diabetics compared with nondiabetics: a meta-analysis. J Diabetes Complications. 2006;20(1):59-68. 10.1016/j.jdiacomp.2005.05.006	287
4	Haubek D, Ennibi OK, Poulsen K, Vaeth M, Poulsen S, Kilian M. Risk of aggressive periodontitis in adolescent carriers of the JP2 clone of Aggregatibacter (Actinobacillus) actinomycetemcomitans in Morocco: a prospective longitudinal cohort study. Lancet. 2008;371(9608):237-242. 10.1016/S0140-6736(08)60135-X	261
5	Ortiz P, Bissada NF, Palomo L, et al. Periodontal therapy reduces the severity of active rheumatoid arthritis in patients treated with or without tumor necrosis factor inhibitors. J Periodontol. 2009;80(4):535-540. 10.1902/jop.2009.080447	244
6	Bouri A Jr, Bissada N, Al-Zahrani MS, Faddoul F, Nouneh I. Width of keratinized gingiva and the health status of the supporting tissues around dental implants. Int J Oral Maxillofac Implants. 2008;23(2):323-326. PMID: 18548930	207
7	Khader YS, Albashaireh ZS, Alomari MA. Periodontal diseases and the risk of coronary heart and cerebrovascular diseases: a meta-analysis. J Periodontol. 2004;75(8):1046-1053. 10.1902/jop.2004.75.8.1046	181
8	AlJehani YA. Risk factors of periodontal disease: review of the literature. Int J Dent. 2014;2014:182513. 10.1155/2014/182513	177
9	Nasajpour A, Ansari S, Rinoldi C, et al. A Multifunctional Polymeric Periodontal Membrane with Osteogenic and Antibacterial Characteristics. Adv Funct Mater. 2018;28(3): 1703437. 10.1002/adfm.201703437	165
10	Mouhyi J, Dohan Ehrenfest DM, Albrektsson T. The peri-implantitis: implant surfaces, microstructure, and physicochemical aspects. Clin Implant Dent Relat Res. 2012;14(2):170-183. 10.1111/j.1708-8208.2009.00244.x	162
11	Khader YS, Ta'ani Q. Periodontal diseases and the risk of preterm birth and low birth weight: a meta-analysis. J Periodontol. 2005;76(2):161-165. 10.1902/jop.2005.76.2.161	158
12	Marouf N, Cai W, Said KN, et al. Association between periodontitis and severity of COVID-19 infection: A case-control study. J Clin Periodontol. 2021;48(4):483-491. 10.1111/jcpe.13435	135
13	Nazir M, Al-Ansari A, Al-Khalifa K, Alhareky M, Gaffar B, Almas K. Global Prevalence of Periodontal Disease and Lack of Its Surveillance. Scientific World Journal. 2020;2020:2146160. 10.1155/2020/2146160	132
14	El-Sharkawy H, Aboelsaad N, Eliwa M, et al. Adjunctive treatment of chronic periodontitis with daily dietary supplementation with omega-3 Fatty acids and low-dose aspirin. J Periodontol. 2010;81(11):1635-1643. 10.1902/jop.2010.090628	132
15	Al-Shammari KF, Al-Khabbaz AK, Al-Ansari JM, Neiva R, Wang HL. Risk indicators for tooth loss due to periodontal disease. J Periodontol. 2005;76(11):1910-1918. 10.1902/jop.2005.76.11.1910	127
16	Fawzy El-Sayed KM, Dörfer CE. Gingival Mesenchymal Stem/Progenitor Cells: A Unique Tissue Engineering Gem. Stem Cells Int. 2016;2016:7154327. 10.1155/2016/7154327	125
17	Sheikh Z, Qureshi J, Alshahrani AM, et al. Collagen based barrier membranes for periodontal guided bone regeneration applications. Odontology. 2017;105(1):1-12. 10.1007/s10266-016-0267-0	106
18	Emmanuel R, Palanisamy S, Chen SM, et al. Antimicrobial efficacy of green synthesized drug blended silver nanoparticles against dental caries and periodontal disease causing microorganisms. Mater Sci Eng C Mater Biol Appl. 2015;56:374-379. 10.1016/j.msec.2015.06.033	103
19	Najeeb S, Zafar MS, Khurshid Z, Zohaib S, Almas K. The Role of Nutrition in Periodontal Health: An Update. Nutrients. 2016;8(9):530. 10.3390/nu8090530	101
20	Haubek D, Ennibi OK, Poulsen K, Poulsen S, Benzarti N, Kilian M. Early-onset periodontitis in Morocco is associated with the highly leukotoxic clone of Actinobacillus actinomycetemcomitans. J Dent Res. 2001;80(6):1580-1583. 10.1177/00220345010800062001	99
21	Tawil G, Younan R, Azar P, Sleilati G. Conventional and advanced implant treatment in the type II diabetic patient: surgical protocol and long-term clinical results. Int J Oral Maxillofac Implants. 2008;23(4):744-752. PMID: 18807573	98
22	Diab-Ladki R, Pellat B, Chahine R. Decrease in the total antioxidant activity of saliva in patients with periodontal diseases. Clin Oral Investig. 2003;7(2):103-107. 10.1007/s00784-003-0208-5	98
23	Javed F, Al-Hezaimi K, Salameh Z, Almas K, Romanos GE. Proinflammatory cytokines in the crevicular fluid of patients with peri-implantitis. Cytokine. 2011;53(1):8-12. 10.1016/j.cyto.2010.08.013	96
24	Khader YS, Bawadi HA, Haroun TF, Alomari M, Tayyem RF. The association between periodontal disease and obesity among adults in Jordan. J Clin Periodontol. 2009;36(1):18-24. 10.1111/j.1600-051X.2008.01345.x	96
25	El-Kamel AH, Ashri LY, Alsarra IA. Micromatricial metronidazole benzoate film as a local mucoadhesive delivery system for treatment of periodontal diseases. AAPS PharmSciTech. 2007;8(3):E75. 10.1208/pt0803075	92
26	Al-Zahrani MS, Borawski EA, Bissada NF. Periodontitis and three health-enhancing behaviors: maintaining normal weight, engaging in recommended level of exercise, and consuming a high-quality diet. J Periodontol. 2005;76(8):1362-1366. 10.1902/jop.2005.76.8.1362	92
27	Mascarenhas P, Gapski R, Al-Shammari K, et al. Clinical response of azithromycin as an adjunct to non-surgical periodontal therapy in smokers. J Periodontol. 2005;76(3):426-436. 10.1902/jop.2005.76.3.426	87
28	Helal O, Göstemeyer G, Krois J, Fawzy El Sayed K, Graetz C, Schwendicke F. Predictors for tooth loss in periodontitis patients: Systematic review and meta-analysis. J Clin Periodontol. 2019;46(7):699-712. 10.1111/jcpe.13118	85
29	Baqain ZH, Moqbel WY, Sawair FA. Early dental implant failure: risk factors. Br J Oral Maxillofac Surg. 2012;50(3):239-243. 10.1016/j.bjoms.2011.04.074	85
30	Hart TC, Hart PS, Michalec MD, et al. Localisation of a gene for prepubertal periodontitis to chromosome 11q14 and identification of a cathepsin C gene mutation. J Med Genet. 2000;37(2):95-101. 10.1136/jmg.37.2.95	84
31	Akram Z, Al-Shareef SA, Daood U, et al. Bactericidal Efficacy of Photodynamic Therapy Against Periodontal Pathogens in Periodontal Disease: A Systematic Review. Photomed Laser Surg. 2016;34(4):137-149. 10.1089/pho.2015.4076	83
32	Gad HA, El-Nabarawi MA, Abd El-Hady SS. Formulation and evaluation of PLA and PLGA in situ implants containing secnidazole and/or doxycycline for treatment of periodontitis. AAPS PharmSciTech. 2008;9(3):878-884. 10.1208/s12249-008-9126-9	82
33	Hewitt C, McCormick D, Linden G, et al. The role of cathepsin C in Papillon-Lefèvre syndrome, prepubertal periodontitis, and aggressive periodontitis. Hum Mutat. 2004;23(3):222-228. 10.1002/humu.10314	82
34	Akram Z, Abduljabbar T, Abu Hassan MI, Javed F, Vohra F. Cytokine Profile in Chronic Periodontitis Patients with and without Obesity: A Systematic Review and Meta-Analysis. Dis Markers. 2016;2016:4801418. 10.1155/2016/4801418	81
35	Chapple IL, Milward MR, Ling-Mountford N, et al. Adjunctive daily supplementation with encapsulated fruit, vegetable and berry juice powder concentrates and clinical periodontal outcomes: a double-blind RCT. J Clin Periodontol. 2012;39(1):62-72. 10.1111/j.1600-051X.2011.01793.x	81
36	Vigolo P, Givani A, Majzoub Z, Cordioli G. Clinical evaluation of small-diameter implants in single-tooth and multiple-implant restorations: a 7-year retrospective study. Int J Oral Maxillofac Implants. 2004;19(5):703-709. PMID: 15508986	81
37	Balto HA. Attachment and morphological behavior of human periodontal ligament fibroblasts to mineral trioxide aggregate: a scanning electron microscope study. J Endod. 2004;30(1):25-29. 10.1097/00004770-200401000-00005	80
38	Akram Z, Hyder T, Al-Hamoudi N, Binshabaib MS, Alharthi SS, Hanif A. Efficacy of photodynamic therapy versus antibiotics as an adjunct to scaling and root planing in the treatment of periodontitis: A systematic review and meta-analysis. Photodiagnosis Photodyn Ther. 2017;19:86-92. 10.1016/j.pdpdt.2017.05.007	79
39	Elkhouli AM. The efficacy of host response modulation therapy (omega-3 plus low-dose aspirin) as an adjunctive treatment of chronic periodontitis (clinical and biochemical study). J Periodontal Res. 2011;46(2):261-268. 10.1111/j.1600-0765.2010.01336.x	79
40	Müller HP, Eger T. Masticatory mucosa and periodontal phenotype: a review. Int J Periodontics Restorative Dent. 2002;22(2):172-183. 10.11607/prd.00.0468	78
41	Guiha R, el Khodeiry S, Mota L, Caffesse R. Histological evaluation of healing and revascularization of the subepithelial connective tissue graft. J Periodontol. 2001;72(4):470-478. 10.1902/jop.2001.72.4.470	78
42	Al-Zahrani MS. Increased intake of dairy products is related to lower periodontitis prevalence. J Periodontol. 2006;77(2):289-294. 10.1902/jop.2006.050082	76
43	Artun J, Grobéty D. Periodontal status of mandibular incisors after pronounced orthodontic advancement during adolescence: a follow-up evaluation. Am J Orthod Dentofacial Orthop. 2001;119(1):2-10. 10.1067/mod.2001.111403	76
44	Ghallab NA. Diagnostic potential and future directions of biomarkers in gingival crevicular fluid and saliva of periodontal diseases: Review of the current evidence. Arch Oral Biol. 2018;87:115-124. 10.1016/j.archoralbio.2017.12.022	75
45	Javed F, Al-Askar M, Al-Hezaimi K. Cytokine profile in the gingival crevicular fluid of periodontitis patients with and without type 2 diabetes: a literature review. J Periodontol. 2012;83(2):156-161. 10.1902/jop.2011.110207	75
46	Bawadi HA, Khader YS, Haroun TF, Al-Omari M, Tayyem RF. The association between periodontal disease, physical activity and healthy diet among adults in Jordan. J Periodontal Res. 2011;46(1):74-81. 10.1111/j.1600-0765.2010.01314.x	74
47	Al-Zahrani MS, Bamshmous SO, Alhassani AA, Al-Sherbini MM. Short-term effects of photodynamic therapy on periodontal status and glycemic control of patients with diabetes. J Periodontol. 2009;80(10):1568-1573. 10.1902/jop.2009.090206	74
48	Kotsakis GA, Javed F, Hinrichs JE, Karoussis IK, Romanos GE. Impact of cigarette smoking on clinical outcomes of periodontal flap surgical procedures: a systematic review and meta-analysis. J Periodontol. 2015;86(2):254-263. 10.1902/jop.2014.140452	73
49	Anand PS, Kamath KP, Anil S. Role of dental plaque, saliva and periodontal disease in Helicobacter pylori infection. World J Gastroenterol. 2014;20(19):5639-5653. 10.3748/wjg.v20.i19.5639	73
50	Al-Zahrani MS, Borawski EA, Bissada NF. Increased physical activity reduces prevalence of periodontitis. J Dent. 2005;33(9):703-710. 10.1016/j.jdent.2005.01.004	73
51	Al-Rasheed A, Scheerens H, Rennick DM, Fletcher HM, Tatakis DN. Accelerated alveolar bone loss in mice lacking interleukin-10. J Dent Res. 2003;82(8):632-635. 10.1177/154405910308200812	72
52	Lang WP, Ronis DL, Farghaly MM. Preventive behaviors as correlates of periodontal health status. J Public Health Dent. 1995;55(1):10-17. 10.1111/j.1752-7325.1995.tb02324.x	71
53	Al-Sowygh ZH, Ghani SMA, Sergis K, Vohra F, Akram Z. Peri-implant conditions and levels of advanced glycation end products among patients with different glycemic control. Clin Implant Dent Relat Res. 2018;20(3):345-351. 10.1111/cid.12584	70
54	Vohra F, Akram Z, Safii SH, et al. Role of antimicrobial photodynamic therapy in the treatment of aggressive periodontitis: A systematic review. Photodiagnosis Photodyn Ther. 2016;13:139-147. 10.1016/j.pdpdt.2015.06.010	70
55	Javed F, Al-Rasheed A, Almas K, Romanos GE, Al-Hezaimi K. Effect of cigarette smoking on the clinical outcomes of periodontal surgical procedures. Am J Med Sci. 2012;343(1):78-84. 10.1097/MAJ.0b013e318228283b	70
56	Fu JH, Lee A, Wang HL. Influence of tissue biotype on implant esthetics. Int J Oral Maxillofac Implants. 2011;26(3):499-508. PMID: 21691596	70
57	Al-Khabbaz AK, Griffin TJ, Al-Shammari KF. Assessment of pain associated with the surgical placement of dental implants. J Periodontol. 2007;78(2):239-246. 10.1902/jop.2007.060032	70
58	Taani DQ, Habashneh R, Hammad MM, Batieha A. The periodontal status of pregnant women and its relationship with socio-demographic and clinical variables. J Oral Rehabil. 2003;30(4):440-445. 10.1046/j.1365-2842.2003.01058.x	70
59	Fawzy El-Sayed KM, Paris S, Becker ST, et al. Periodontal regeneration employing gingival margin-derived stem/progenitor cells: an animal study. J Clin Periodontol. 2012;39(9):861-870. 10.1111/j.1600-051X.2012.01904.x	69
60	Elsyad MA, Gebreel AA, Fouad MM, Elshoukouki AH. The clinical and radiographic outcome of immediately loaded mini implants supporting a mandibular overdenture. A 3-year prospective study. J Oral Rehabil. 2011;38(11):827-834. 10.1111/j.1365-2842.2011.02213.x	69
61	Liu X, He X, Jin D, et al. A biodegradable multifunctional nanofibrous membrane for periodontal tissue regeneration. Acta Biomater. 2020;108:207-222. 10.1016/j.actbio.2020.03.044	68
62	Al Amri MD, Kellesarian SV, Al-Kheraif AA, Malmstrom H, Javed F, Romanos GE. Effect of oral hygiene maintenance on HbA1c levels and peri-implant parameters around immediately-loaded dental implants placed in type-2 diabetic patients: 2 years follow-up. Clin Oral Implants Res. 2016;27(11):1439-1443. 10.1111/clr.12758	68
63	Sakka S, Coulthard P. Implant failure: etiology and complications. Med Oral Patol Oral Cir Bucal. 2011;16(1):e42-e44. 10.4317/medoral.16.e42	68
64	Al-Otaibi M, Al-Harthy M, Söder B, Gustafsson A, Angmar-Månsson B. Comparative effect of chewing sticks and toothbrushing on plaque removal and gingival health. Oral Health Prev Dent. 2003;1(4):301-307. 10.3290/j.ohpd.a8667	68
65	Nasra MM, Khiri HM, Hazzah HA, Abdallah OY. Formulation, in-vitro characterization and clinical evaluation of curcumin in-situ gel for treatment of periodontitis. Drug Deliv. 2017;24(1):133-142. 10.1080/10717544.2016.1233591	67
66	Habashneh RA, Khader YS, Alhumouz MK, Jadallah K, Ajlouni Y. The association between inflammatory bowel disease and periodontitis among Jordanians: a case-control study. J Periodontal Res. 2012;47(3):293-298. 10.1111/j.1600-0765.2011.01431.x	67
67	Elsyad MA, Al-Mahdy YF, Fouad MM. Marginal bone loss adjacent to conventional and immediate loaded two implants supporting a ball-retained mandibular overdenture: a 3-year randomized clinical trial. Clin Oral Implants Res. 2012;23(4):496-503. 10.1111/j.1600-0501.2011.02173.x	67
68	Amarante ES, Leknes KN, Skavland J, Lie T. Coronally positioned flap procedures with or without a bioabsorbable membrane in the treatment of human gingival recession. J Periodontol. 2000;71(6):989-998. 10.1902/jop.2000.71.6.989	67
69	Al Habashneh R, Khader YS, Salameh S. Use of the Arabic version of Oral Health Impact Profile-14 to evaluate the impact of periodontal disease on oral health-related quality of life among Jordanian adults. J Oral Sci. 2012;54(1):113-120. 10.2334/josnusd.54.113	66
70	Romanos GE, Javed F, Delgado-Ruiz RA, Calvo-Guirado JL. Peri-implant diseases: a review of treatment interventions. Dent Clin North Am. 2015;59(1):157-178. 10.1016/j.cden.2014.08.002	65
71	Javed F, Abduljabbar T, Vohra F, Malmstrom H, Rahman I, Romanos GE. Comparison of Periodontal Parameters and Self-Perceived Oral Symptoms Among Cigarette Smokers, Individuals Vaping Electronic Cigarettes, and Never-Smokers. J Periodontol. 2017;88(10):1059-1065. 10.1902/jop.2017.170197	64
72	Akram Z, Abduljabbar T, Sauro S, Daood U. Effect of photodynamic therapy and laser alone as adjunct to scaling and root planing on gingival crevicular fluid inflammatory proteins in periodontal disease: A systematic review. Photodiagnosis Photodyn Ther. 2016;16:142-153. 10.1016/j.pdpdt.2016.09.004	64
73	Alwaeli HA, Al-Khateeb SN, Al-Sadi A. Long-term clinical effect of adjunctive antimicrobial photodynamic therapy in periodontal treatment: a randomized clinical trial. Lasers Med Sci. 2015;30(2):801-807. 10.1007/s10103-013-1426-y	63
74	Ababneh KT, Abu Hwaij ZM, Khader YS. Prevalence and risk indicators of gingivitis and periodontitis in a multi-centre study in North Jordan: a cross sectional study. BMC Oral Health. 2012;12:1. 10.1186/1472-6831-12-1	63
75	Abou Sulaiman AE, Shehadeh RM. Assessment of total antioxidant capacity and the use of vitamin C in the treatment of non-smokers with chronic periodontitis. J Periodontol. 2010;81(11):1547-1554. 10.1902/jop.2010.100173	63
76	Al Hamad KQ, Azar WZ, Alwaeli HA, Said KN. A clinical study on the effects of cordless and conventional retraction techniques on the gingival and periodontal health. J Clin Periodontol. 2008;35(12):1053-1058. 10.1111/j.1600-051X.2008.01335.x	63
77	Fawzy El-Sayed KM, Mekhemar MK, Beck-Broichsitter BE, et al. Periodontal regeneration employing gingival margin-derived stem/progenitor cells in conjunction with IL-1ra-hydrogel synthetic extracellular matrix. J Clin Periodontol. 2015;42(5):448-457. 10.1111/jcpe.12401	62
78	Müller HP, Könönen E. Variance components of gingival thickness. J Periodontal Res. 2005;40(3):239-244. 10.1111/j.1600-0765.2005.00798.x	62
79	Darout IA, Albandar JM, Skaug N, Ali RW. Salivary microbiota levels in relation to periodontal status, experience of caries and miswak use in Sudanese adults. J Clin Periodontol. 2002;29(5):411-420. 10.1034/j.1600-051x.2002.290505.x	61
80	Al-Otaibi M, Al-Harthy M, Gustafsson A, Johansson A, Claesson R, Angmar-Månsson B. Subgingival plaque microbiota in Saudi Arabians after use of miswak chewing stick and toothbrush. J Clin Periodontol. 2004;31(12):1048-1053. 10.1111/j.1600-051X.2004.00618.x	60
81	Mokeem SA, Molla GN, Al-Jewair TS. The prevalence and relationship between periodontal disease and pre-term low birth weight infants at King Khalid University Hospital in Riyadh, Saudi Arabia. J Contemp Dent Pract. 2004;5(2):40-56. PMID: 15150633	60
82	Abdelaziz D, Hefnawy A, Al-Wakeel E, El-Fallal A, El-Sherbiny IM. New biodegradable nanoparticles-in-nanofibers based membranes for guided periodontal tissue and bone regeneration with enhanced antibacterial activity. J Adv Res. 2020;28:51-62. 10.1016/j.jare.2020.06.014	59
83	emlali A, Witoled C, Alanazi M, Rouabhia M. Whole cigarette smoke increased the expression of TLRs, HBDs, and proinflammory cytokines by human gingival epithelial cells through different signaling pathways. PLoS One. 2012;7(12):e52614. 10.1371/journal.pone.0052614	59
84	Oghli AA, Steveling H. Ridge preservation following tooth extraction: a comparison between atraumatic extraction and socket seal surgery. Quintessence Int. 2010;41(7):605-609. PMID: 20614049	59
85	El-Sayed KM, Paris S, Graetz C, et al. Isolation and characterisation of human gingival margin-derived STRO-1/MACS(+) and MACS(-) cell populations. Int J Oral Sci. 2015;7(2):80-88. 10.1038/ijos.2014.41	58
86	Lee A, Fu JH, Wang HL. Soft tissue biotype affects implant success. Implant Dent. 2011;20(3):e38-e47. 10.1097/ID.0b013e3182181d3d	58
87	Khader Y, Khassawneh B, Obeidat B, et al. Periodontal status of patients with metabolic syndrome compared to those without metabolic syndrome. J Periodontol. 2008;79(11):2048-2053. 10.1902/jop.2008.080022	58
88	Akram Z, Shafqat SS, Niaz MO, Raza A, Naseem M. Clinical efficacy of photodynamic therapy and laser irradiation as an adjunct to open flap debridement in the treatment of chronic periodontitis: A systematic review and meta-analysis. Photodermatol Photoimmunol Photomed. 2020;36(1):3-13. 10.1111/phpp.12499	57
89	Banu S, Jabir NR, Mohan R, et al. Correlation of Toll-like receptor 4, interleukin-18, transaminases, and uric acid in patients with chronic periodontitis and healthy adults. J Periodontol. 2015;86(3):431-439. 10.1902/jop.2014.140414	57
90	Alshouibi EN, Kaye EK, Cabral HJ, Leone CW, Garcia RI. Vitamin D and periodontal health in older men. J Dent Res. 2013;92(8):689-693. 10.1177/0022034513495239	57
91	Khader Y, Al-shishani L, Obeidat B, et al. Maternal periodontal status and preterm low birth weight delivery: a case-control study. Arch Gynecol Obstet. 2009;279(2):165-169. 10.1007/s00404-008-0696-2	57
92	ALHarthi SSY, Natto ZS, Midle JB, Gyurko R, O'Neill R, Steffensen B. Association between time since quitting smoking and periodontitis in former smokers in the National Health and Nutrition Examination Surveys (NHANES) 2009 to 2012. J Periodontol. 2019;90(1):16-25. 10.1002/JPER.18-0183	56
93	BinShabaib M, ALHarthi SS, Akram Z, et al. Clinical periodontal status and gingival crevicular fluid cytokine profile among cigarette-smokers, electronic-cigarette users and never-smokers. Arch Oral Biol. 2019;102:212-217. 10.1016/j.archoralbio.2019.05.001	55
94	Abduljabbar T, Vohra F, Javed F, Akram Z. Antimicrobial photodynamic therapy adjuvant to non-surgical periodontal therapy in patients with diabetes mellitus: A meta-analysis. Photodiagnosis Photodyn Ther. 2017;17:138-146. 10.1016/j.pdpdt.2016.11.008	55
95	Elsyad MA, Elsaih EA, Khairallah AS. Marginal bone resorption around immediate and delayed loaded implants supporting a locator-retained mandibular overdenture. A 1-year randomised controlled trial. J Oral Rehabil. 2014;41(8):608-618. 10.1111/joor.12182	55
96	Casado PL, Otazu IB, Balduino A, de Mello W, Barboza EP, Duarte ME. Identification of periodontal pathogens in healthy periimplant sites. Implant Dent. 2011;20(3):226-235. 10.1097/ID.0b013e3182199348	55
97	Al Habashneh R, Alchalabi H, Khader YS, Hazza'a AM, Odat Z, Johnson GK. Association between periodontal disease and osteoporosis in postmenopausal women in Jordan. J Periodontol. 2010;81(11):1613-1621. 10.1902/jop.2010.100190	55
98	El-Sharkawy HM, Anees MM, Van Dyke TE. Propolis Improves Periodontal Status and Glycemic Control in Patients With Type 2 Diabetes Mellitus and Chronic Periodontitis: A Randomized Clinical Trial. J Periodontol. 2016;87(12):1418-1426. 10.1902/jop.2016.150694	54
99	Javed F, Al-Kheraif AA, Rahman I, et al. Comparison of Clinical and Radiographic Periodontal Status Between Habitual Water-Pipe Smokers and Cigarette Smokers. J Periodontol. 2016;87(2):142-147. 10.1902/jop.2015.150235	54
100	Majeed ZN, Philip K, Alabsi AM, Pushparajan S, Swaminathan D. Identification of Gingival Crevicular Fluid Sampling, Analytical Methods, and Oral Biomarkers for the Diagnosis and Monitoring of Periodontal Diseases: A Systematic Review. Dis Markers. 2016;2016:1804727. 10.1155/2016/1804727	54
